# Risk Model and Immune Signature of m7G-Related lncRNA Based on Lung Adenocarcinoma

**DOI:** 10.3389/fgene.2022.907754

**Published:** 2022-06-08

**Authors:** Chuanhao Zhang, Dong Zhou, Zhe Wang, Zaishuang Ju, Jiabei He, Genghao Zhao, Ruoyu Wang

**Affiliations:** ^1^ Graduate School of Dalian Medical University, Dalian, China; ^2^ Departement of Medical Oncology, Affiliated Zhongshan Hospital of Dalian University, Dalian, China

**Keywords:** lung adenocarcinoma (AC), m7G, lncRNA, model, immune signature, treatment

## Abstract

Lung cancer is a major cause of cancer-related deaths globally, with a dismal prognosis. N7-methylguanosine (m7G) is essential for the transcriptional phenotypic modification of messenger RNA (mRNA) and long noncoding RNA (lncRNA). However, research on m7G-related lncRNAs involved in lung adenocarcinoma (LUAD) regulation is still limited. Herein, we aim to establish a prognostic model of m7G-related lncRNAs and investigate their immune properties. Eight prognostic m7G-related lncRNAs were identified using univariate Cox analysis. Six m7G-related lncRNAs were identified using LASSO-Cox regression analysis to construct risk models, and all LUAD patients in The Cancer Genome Atlas (TCGA) cohort was divided into low-risk and high-risk subgroups. The accuracy of the model was verified by Kaplan-Meier analysis, time-dependent receiver operating characteristic, principal component analysis, independent prognostic analysis, nomogram, and calibration curve. Further studies were conducted on the gene set enrichment and disease ontology enrichment analyses. The gene set enrichment analysis (GSEA) revealed that the high-risk group enriched for cancer proliferation pathways, and the enrichment analysis of disease ontology (DO) revealed that lung disease was enriched, rationally explaining the superiority of the risk model. Finally, we found that the low-risk group had higher immune infiltration and checkpoint expression. It can be speculated that the low-risk group has a better effect on immunotherapy. Susceptibility to antitumor drugs in different risk subgroups was assessed, and it found that the high-risk group showed high sensitivity to first-line treatment drugs for non-small cell lung cancer. In conclusion, a risk model based on 6 m7G-related lncRNAs can not only predict the overall survival (OS) rate of LUAD patients but also guide individualized treatment for these patients.

## Introduction

Lung cancer is the second most common type of cancer worldwide and the leading cause of cancer mortality, accounting for approximately 11.4% of diagnosed cancers and 18.0% of deaths (Sung et al., 2021). Currently, the 5-years survival rate for lung cancer is still very low, only 10–20% in most countries (Allemani et al., 2018). Lung adenocarcinoma (LUAD) is the most commonly diagnosed subtype of lung cancer, accounting for approximately 40% of all cases (Travis et al., 2015). With the development of surgery, radiotherapy, chemotherapy, targeted therapy, and immunotherapy, the 5-years survival rate of lung cancer has improved, but the performance remains unsatisfactory. There is an urgent need to develop a convenient and fast prognostic model that can accurately judge patient prognosis and guide individualized treatment, which could be very useful for both patients and clinicians.

In humans, the methyltransferase like 1 (METTL1)/WD repeat domain 4 (WDR4) complex catalyzes N7-methylguanosine, one of the most common tRNA modifications in the tRNA variable loop (Alexandrov et al., 2005; Lin et al., 2018). METTL1 is an m7G catalytic enzyme and WDR4 is important in the methyltransferase complex stabilization (Alexandrov et al., 2002). Recently, it was found that METTL1 and WDR4 were significantly up-regulated in lung cancer tissues and played an oncogenic role in lung cancer via mediating m7G tRNA modification and modulated the translation of mRNAs, especially METTL1-mediated m7G tRNA modification and m7G codon usage promoted mRNA translation and lung cancer progression (Ma et al., 2021). This suggests that METTL1 and WDR4 may play a significant role in tumor progression. Therefore, screening m7G-related genes is essential.

Long non-coding RNAs (lncRNAs) are defined as non-coding RNAs of more than 200 nucleotides in length. They are not generally considered to encode proteins but are involved in the regulation of different levels (epigenetic regulation, transcriptional regulation, and post-transcriptional regulation) of genes encoding proteins in the form of RNA (Juhling et al., 2009; Spizzo et al., 2012). Several lncRNAs, including ferroptosis-related lncRNAs (Chen et al., 2022), pyroptosis-related lncRNAs (Xu et al., 2022), and autophagy-related lncRNAs (Luo et al., 2022), have recently been implicated in prognosis in cancer patients, while m7G-related lncRNAs have rarely been reported.

Herein, we identified 6 prognostic risk models of m7G-related lncRNAs and the correlation between the risk model and immune characteristics. As expected, our model well predicted survival in LUAD patients and showed greater efficacy in terms of immune cell invasion and immune checkpoint expression.

## Materials and Methods

### Data Set

RNA sequencing data and associated clinical characteristics of 594 LUAD patients were extracted from The Cancer Genome Atlas (TCGA) database, including 59 normal tissues and 535 LUAD tissues. Forty m7G-related genes were obtained from the gene set enrichment analysis (GSEA) website (http://www.gsea-msigdb.org/gsea/login.jsp) and published articles. Patients lacking clinical information were deleted from subsequent analyses.

### Selection of m7G-Related lncRNAs

LncRNAs were screened from 594 patients with LUAD using Strawberry Perl (version 5.30.0). A total of 2093 m7G-related lncRNAs were identified using the limma R package with the following criteria: Pearson correlation coefficient >0.4 and *p* < 0.001. A total of 990 differentially expressed lncRNAs (DELs) were identified in normal lung tissues and LUAD tissues with the following criteria: log_2_ fold change (FC) > 1 and false discovery rate <0.05.

### Development and Validation of m7G-Related lncRNA Prognostic Model

To rigorously screen out prognostic lncRNAs, the *p*-value was set to 0.01 and univariate Cox analysis was used to identify prognostic lncRNAs. Next, the TCGA cohort was randomly divided into a training and a validation group, each accounting for 50%. Based on these prognostic lncRNAs, Lasso-Cox regression analysis was used to select genes to minimize the risk of overfitting and a risk prediction model was constructed. The risk score was calculated using the following formula:

$$risk\;score=\sum_{i=1}^{n}\left(coef_{i}*\exp r_{i}\right)$$ (1)

where coef_i_ represents the coefficients of each lncRNA and expr_i_ represents the expression level of each lncRNA. Based on the median value of the risk score, patients were divided into low-risk and high-risk groups. Survival curves were drawn between low-risk and high-risk groups using the survival and survminer packages of the R software. The stability of the risk score was performed using the validation group. Clinical information (including age, gender, and stage) of TCGA-LUAD patients was extracted and combined with the risk score for univariate and multivariate Cox regression analysis to evaluate whether the risk score is an independent prognostic factor for overall survival (OS), and compared predictive results of different factors using receiver operating characteristic (ROC) curve analysis.

### Nomogram and Calibration

The rms R package was utilized to construct nomograms. Calibration curves were used to quantify the agreement between the predicted and the actual results for 1-, 3-, and 5-years survival rates.

### Gene Set Enrichment Analysis

Kyoto Encyclopedia of Genes and Genomes (KEGG) pathway analysis was performed, and significantly enriched pathways in different risk subgroups were identified using GSEA software (*p* < 0.05 and FDR <0.25).

### Assessment of Immune Cell Infiltration and Immune Checkpoints

All TCGA tumor immune cell infiltration files were downloaded from TIMER 2.0 and the correlation between the explored immune infiltrating cells and the risk score was analyzed using limma, scales, ggplot2 and ggtext R packages. Additionally, immune cell infiltration, immune-related signaling pathways, tumor microenvironment (TME) scores, and immune checkpoints were compared between low-risk and high-risk groups using the ggpubr package.

### Prediction of Drug Susceptibility

The pRRophetic R package was utilized to predict the half-maximal inhibitory concentration (IC_50_) value of cancer drugs in different risk subgroups, which represents the effectiveness of a substance in inhibiting a specific biological or biochemical process.

### Statistical Analysis

All statistical analyses were performed using R software (version 4.0.4). The Wilcoxon signed-rank test was used to investigate differences in the composition of immune infiltrating cells. The correlation between m7G-related genes and m7G-related lncRNAs was investigated using Spearman correlation analysis. Kaplan-Meier analysis was used to estimate survival curves. *p* values <0.05 (∗), 0.01 (∗∗), and 0.001 (∗∗∗) were considered statistically significant.

## Results

### Workflow of Study

**FIGURE 1:**
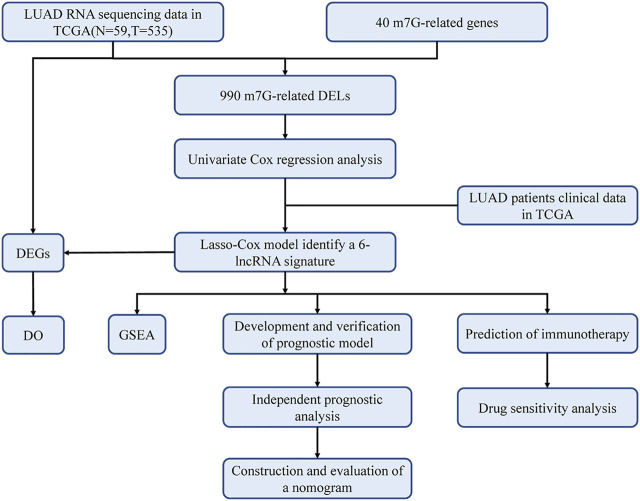
Workflow of this study. The TCGA database was utilized to screen 990 differentially expressed lncRNAs (DELs) in LUAD, which were analyzed with LASSO-COX regression to develop a prognostic model for m7G-related lncRNAs. The prognostic model had been validated in multiple ways and proved to be stable and reliable. Therefore, based on this model, we also performed disease ontology enrichment analysis (DO), gene set enrichment analysis (GSEA), immune-related analysis and drug sensitivity analysis to determine the potential function of prognostic signatures.

The study flowchart is shown in Figure 1. The precise procedure is as follows: First, we obtained RNA sequencing from the TCGA database for 594 lung adenocarcinoma patients, as well as 40 m7G-associated genes from the GSEA database and relevant literature. Furthermore, a 6-lncRNA prognostic model was developed, and its stability was validated using multiple techniques. Finally, GSEA and DO enrichment analysis validated the superiority of the model, while immunological correlation analysis and drug sensitivity analysis extended on the idea of clinical treatment.

### Identification of Differentially Expressed m7G-Related lncRNAs

**FIGURE 2:**
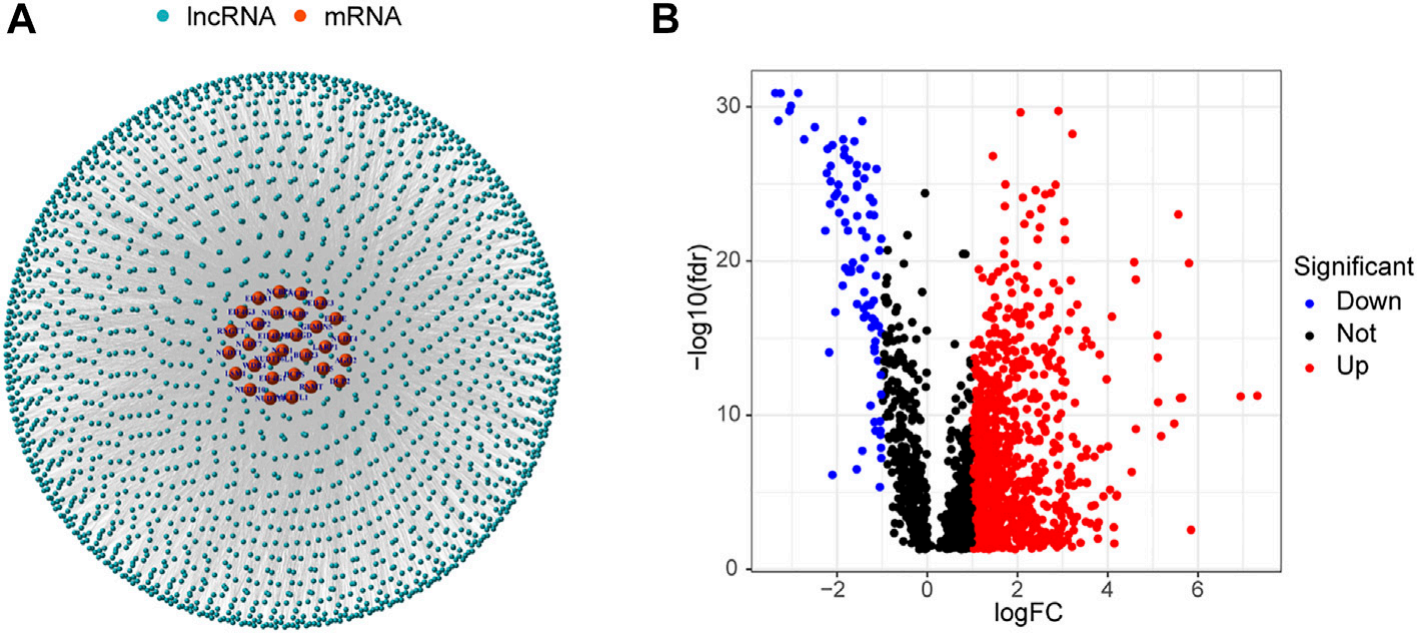
Identification of m7G-related lncRNAs in LUAD patients. **(A)** Co-expression network of m7G-related lncRNAs and mRNAs. **(B)** 990 differentially expressed lncRNAs.

Data for 594 LUAD samples were obtained from the TCGA database, and 14,056 lncRNAs and 19,573 mRNAs were detected. Forty m7G-related genes were obtained from published articles and the GSEA website (Letoquart et al., 2014; Trotman and Schoenberg, 2019; Galloway et al., 2021; Ma et al., 2021). The co-expression network between m7G-related genes and lncRNAs is shown in Figure 2A. A total of 990 DELs were screened from 59 normal tissues and 535 LUAD tissues (|Log_2_ FC| > 1 and *p* < 0.05). Of these, 903 lncRNAs were up-regulated and 87 were down-regulated (Figure 2B).

### Development and Validation of Prognostic Gene Models

**FIGURE 3:**
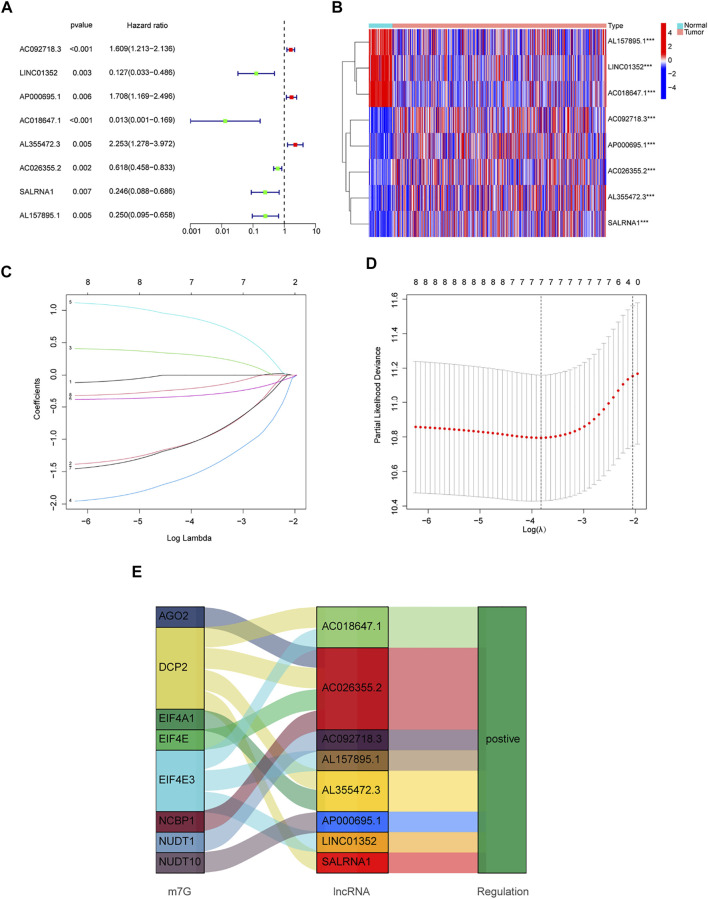
Development of a new prognostic model for m7G-related lncRNAs in LUAD. **(A)** 8 prognosis-related lncRNAs screened by univariate Cox regression analysis (*p* < 0.01). **(B)** Differential expression of prognosis-related lncRNAs in lung normal tissues and adenocarcinoma tissues. **(C)** LASSO coefficient distribution of 8 m7G-related lncRNAs. **(D)** The tuning parameter (λ) in the LASSO model is chosen by the minimum criterion. **(E)** The Sankey diagram depicts the detailed connections between eight prognosis-related lncRNAs and m7G-related genes. *p < 0.05, **p < 0.01, ***p < 0.001.

Patients from the TCGA-LUAD database were randomly split into two groups: a training set and a validation set. To strictly screen prognosis-related DELs, the *p*-value was set to 0.01, and performed univariate Cox regression analysis was performed on the training group. Eight prognosis-related lncRNAs met the conditions, including AC092718.3, LINC01352, AP000695.1, AC018647.1, AL355472.3, AC026355.2, SALRNA1 and AL157895.1 (Figure 3A). These prognosis-related lncRNAs are shown in Figure 3B. Furthermore, these lncRNAs were positively regulated by corresponding genes in the Sankey diagram (Figure 3E). LASSO regression analysis was then performed on these prognosis-associated lncRNAs. Cross-validation was also performed to obtain the best λ value from the smallest partial likelihood bias (Figures 3C,D), to further identify lncRNAs significantly associated with prognosis in LUAD patients. Moreover, multivariate Cox regression analysis was used to screen six prognosis-related lncRNAs and calculate the respective coefficients of these lncRNAs. Finally, six candidates, including LINC01352, AP000695.1, AC018647.1, AL355472.3, AC026355.2, SALRNA1, were selected to construct a risk model. The risk score was calculated using the following formula: LINC01352∗(-1.42486) + AP000695.1∗(0.37854) + AC018647.1∗(-2.19905) + AL355472.3∗(1.05547) + AC026355.2∗(-0.38520) + SALRNA1∗(-1.39428).

**FIGURE 4:**
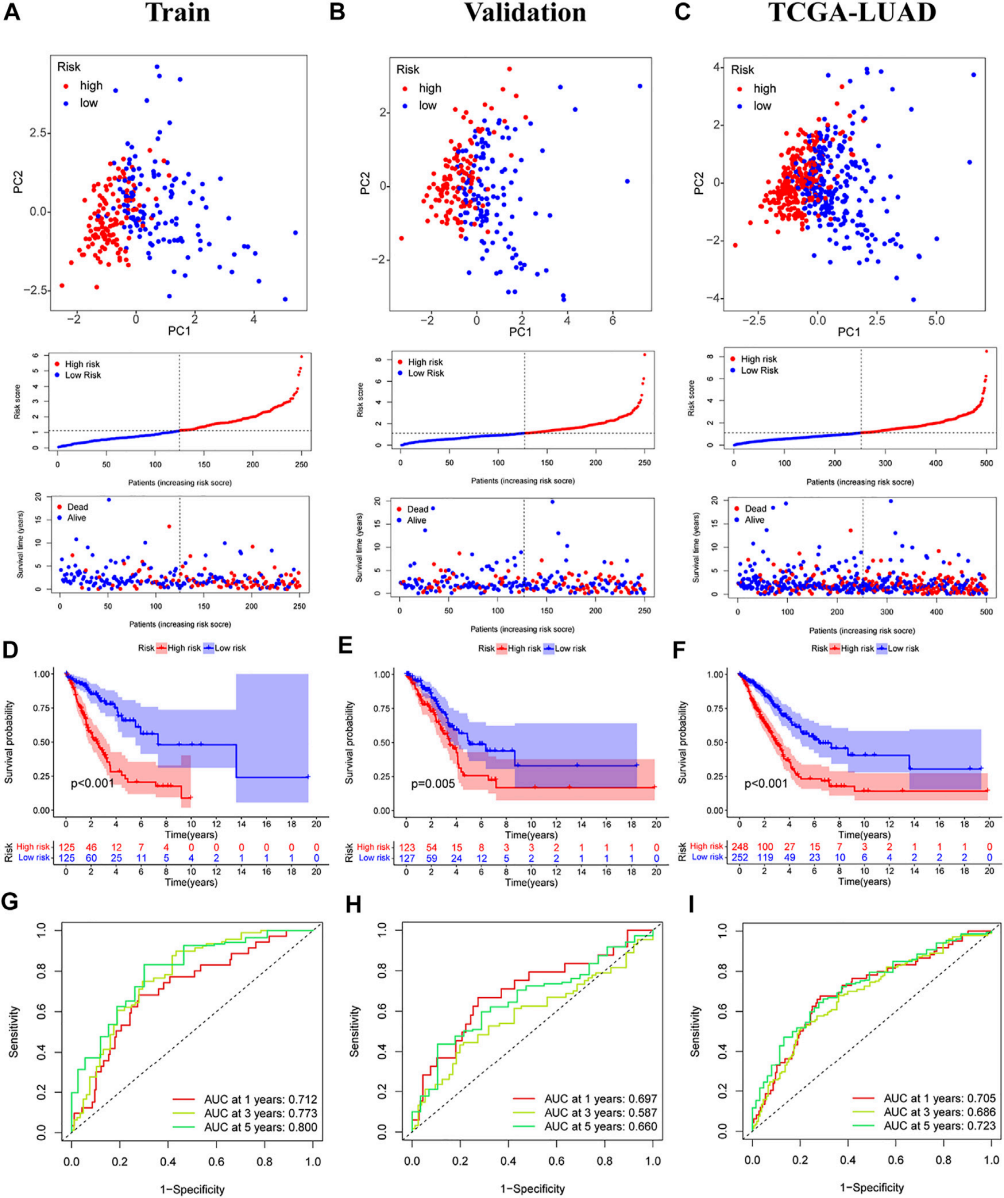
Validation of prognostic models for six m7G-related lncRNAs. **(A–C)** Principal component analysis, risk score distribution, and survival status distribution for training, validation, and TCGA-LUAD. **(D–F)** Kaplan-Meier curves of training group, validation group and TCGA-LUAD at different risk groups. **(G–I)** ROC curves for 1 year, 3 years and 5 years.

The median score was calculated based on the above formula, and the TCGA-LUAD cohort, training group, and validation group were classified into low-risk and high-risk subgroups, and the principal component analysis, risk score distribution, and survival status distribution were visualized, respectively (Figures 4A–C). The results revealed that the sample distribution of the two risk groups was reasonable. Kaplan-Meier survival analysis showed that the OS was shorter in the high-risk group than in the low-risk group (Figures 4D–F).

### Independent Prognostic Value of Risk Models

**FIGURE 5:**
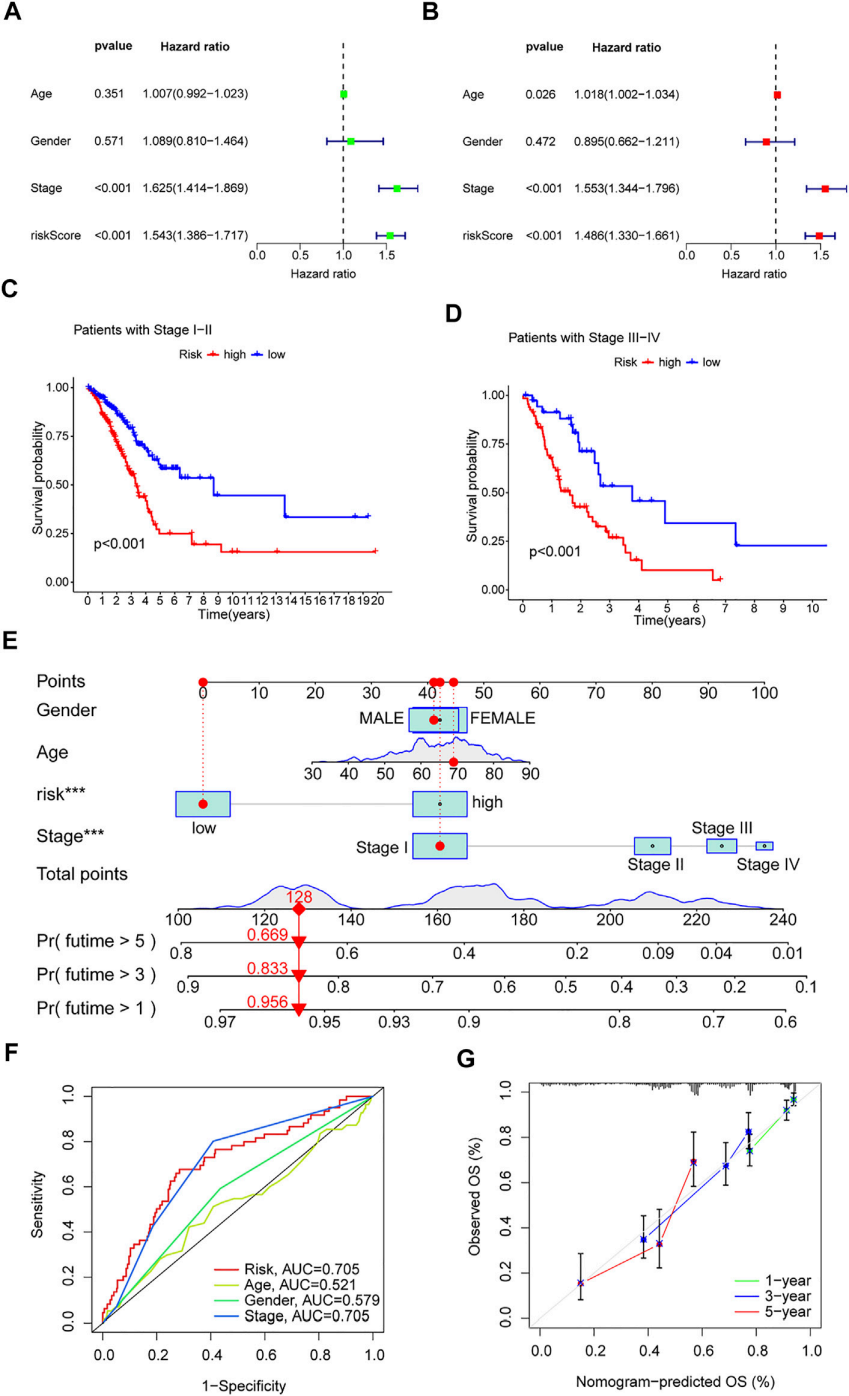
Clinical value of risk characteristics in TCGA-LUAD. **(A)** Univariate Cox regression analysis of risk scores and clinical factors. **(B)** Multivariate Cox regression analysis of risk scores and clinical factors. **(C,D)** Pathological stage was stratified between low- and high-risk groups in the entire collection. **(E)** Nomogram combining gender, age, stage, and risk score predicts 1-, 3-, and 5-years overall survival. **(F)** Clinicopathological features and the predictive accuracy of risk models. **(G)** Calibration curves test the agreement between actual and predicted results at 1, 3, and 5 years. *p < 0.05, **p < 0.01, ***p < 0.001.

Univariate and multivariate Cox regression analyses were performed on the TCGA-LUAD cohort to evaluate the accuracy of the risk model and determine whether risk score could serve as an independent prognostic factor for patient survival. Univariate Cox regression analysis showed that both the risk score and the stage were significantly related to the prognosis of the patient (Figure 5A). After controlling for other confounding factors, multivariate analysis revealed that risk score and stage were independent prognostic factors (Figure 5B). To expand the applicability of the risk model, the stage was divided into two subgroups: early-stage (Stage I and Stage II) and late-stage (Stage III and Stage IV). The survival curves are shown in Figures 5C,D. Patients with advanced Stage had a very poor prognosis, which is completely consistent with the clinical data.

A time-dependent ROC curve was generated in the TCGA-LUAD cohort, and the area under the curve (AUC) reached 0.705, 0.686, and 0.723 at 1, 3, and 5 years, respectively (Figure 4I). In addition, ROC curves confirmed that the risk signature had better prognostic accuracy compared with other clinicopathological features (Figure 5F). Time-dependent ROC curves also showed excellent predictive power in both the training and validation sets (Figures 4G,H).

### Construction of Nomogram

Based on the TCGA-LUAD cohort, risk scores and clinical factors were integrated to create a nomogram (Figure 5E) to improve the predictive power of survival in LUAD patients. Calibration plots for 1-, 3- and 5-years OS revealed good agreement between nomogram prediction and actual observations (Figure 5G).

### GSEA and DO

**FIGURE 6:**
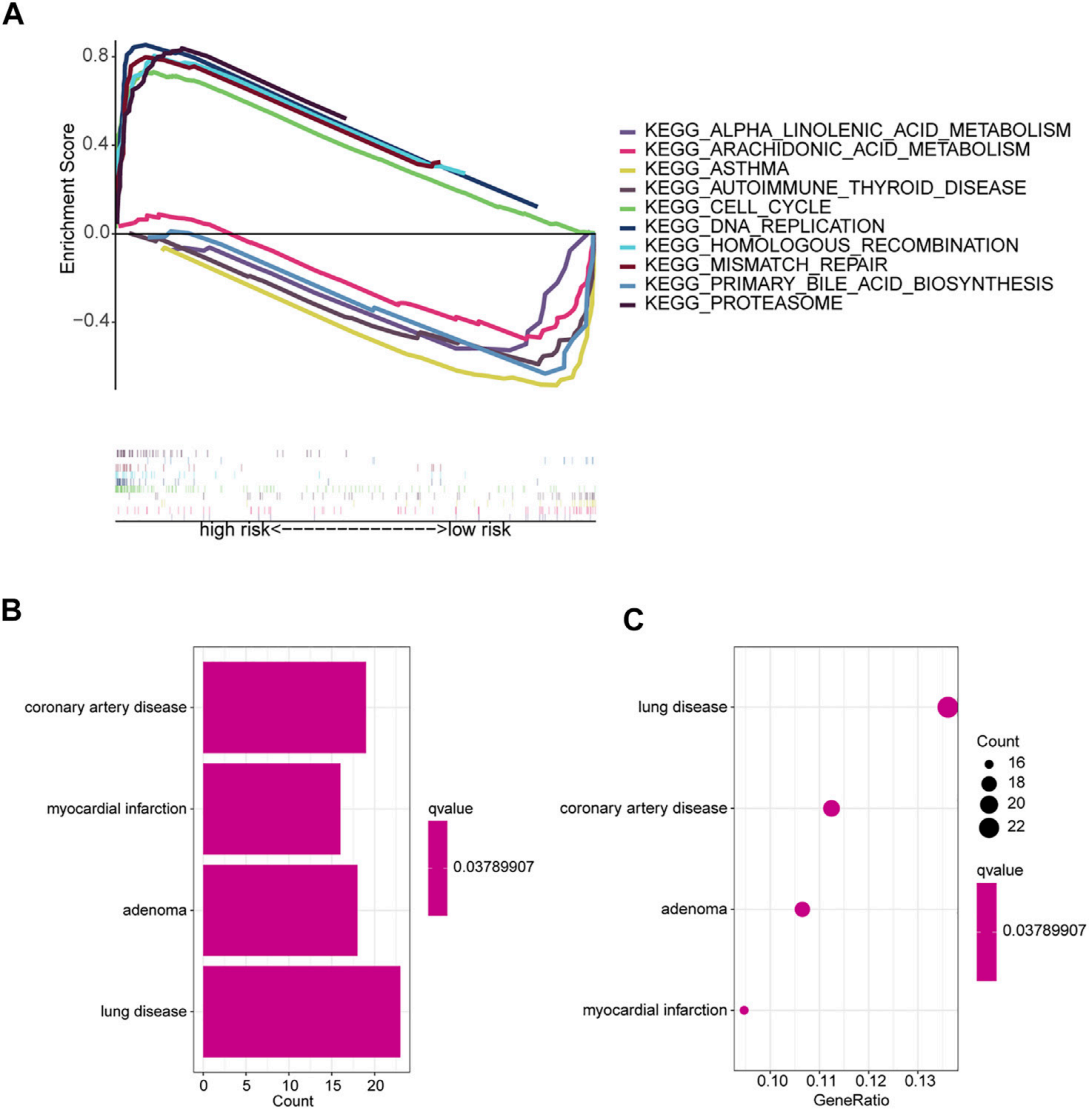
Enrichment analysis of different risk subgroups. **(A)** Five pathways were significantly enriched in each of the high-risk and low-risk groups. **(B,C)** DO enrichment analysis of DEGs based on different risk subgroups.

GSEA software was used to explore KEGG pathways in the entire collection to investigate differences in signaling pathways in different risk subgroups. It was found that pathways related to cancer proliferation, such as cell cycle, DNA replication, mismatch repair, proteasome, homologous recombination, etc., were enriched in the high-risk groups. In addition, the low-risk group was mainly enriched in pathways such as autoimmune thyroid disease, asthma, primary bile acid biosynthesis, arachidonic acid metabolism, and alpha linolenic acid metabolism (Figure 6A). The majority of enriched pathways in the high-risk group were closely related to radiotherapy (Azzam et al., 2012; Haro et al., 2012). Hence, we speculate that radiotherapy may have unexpected effects on patients in the high-risk group, providing foundations for future research directions. Immune-related pathways were enriched in the low-risk group, implying that the low-risk group may be closely related to immune characteristics. Disease differences of differentially expressed genes (DEGs) between the two risk subgroups were further investigated. First, DEGs (|Log_2_ FC| > 1 and *p* < 0.05) between the two risk subgroups were screened, followed by enrichment analysis of disease ontology (DO). DEGs were enriched in lung diseases, adenoma, coronary artery disease, and myocardial infarction (Yu et al., 2012; Yu et al., 2015). This confirms once again that our risk model is very superior. Surprisingly, DEGs are also involved in coronary artery disease and myocardial infarction (Figures 6B,C).

### Relationship Between Risk Model and Immune Characteristics

**FIGURE 7:**
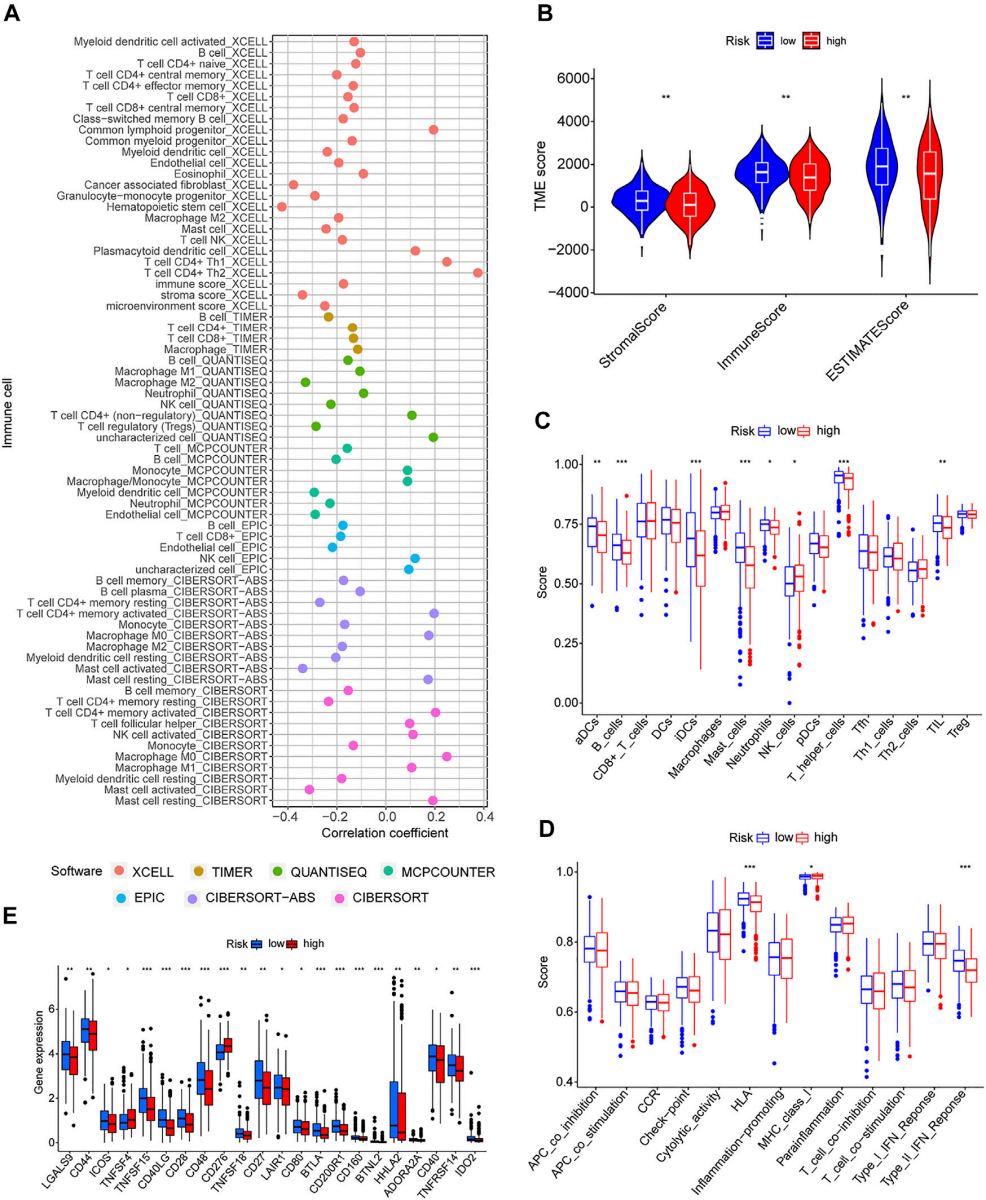
Immune signatures of different risk groups. **(A)** Correlation between risk scores and immune cells. **(B)** Comparison of immune-related scores between low-risk and high-risk groups. **(C,D)** Enrichment scores for 16 immune cells and 13 immune-related pathways. **(E)** Differences in the expression of 22 checkpoints in different risk groups. *p < 0.05, **p < 0.01, ***p < 0.001.

Because GSEA revealed that the low-risk group was enriched in immune-related pathways, we hypothesized that the m7G-related lncRNA-based risk model was strongly tied to immunity. Therefore, the relationship between the risk model and the immunological signature was investigated further. The relationship between immune cells and the risk score is shown in the bubble diagram. The majority of immune cells were negatively correlated with the risk score, especially hematopoietic stem cells of XCELL, tumor-related fibroblasts, stroma score, granulocyte-monocyte progenitor cells, and activated mast cells of CIBERSORT-ABS, resting memory CD4 + T cells, M2 macrophages, and Treg cells of QUANTISEQ and myeloid dendritic cells and endothelial cells of MCPCOUNTER (Figure 7A). Moreover, single sample gene set enrichment analysis (ssGSEA) was used to examine the enrichment fraction of 16 different types of immune cells as well as the activity of 13 different immune-related pathways. Interestingly, the low-risk group had more immune cell infiltration, particularly activated dendritic cells, B cells, immature dendritic cells, mast cells, neutrophils, T helper cells, and tumor-infiltrating lymphocytes (Figure 7C). The activity of type 2 interferon signaling pathway and human leukocyte antigen was higher in the low-risk group than in the high-risk group (Figure 7D). Differential analysis was used to detect differences in the tumor microenvironment between the high-risk and low-risk groups, and the results showed that the low-risk group had higher immune, stromal, and estimate scores (Figure 7B). It is possible to conclude that the low-risk group had greater immune cell infiltration and lower tumor purity. Furthermore, most immune checkpoints were highly expressed in low-risk groups (Figure 7E). Therefore, low-risk patients may benefit more from immune checkpoint inhibitor therapy in our risk model.

### Clinical Application of Risk Model

**FIGURE 8:**
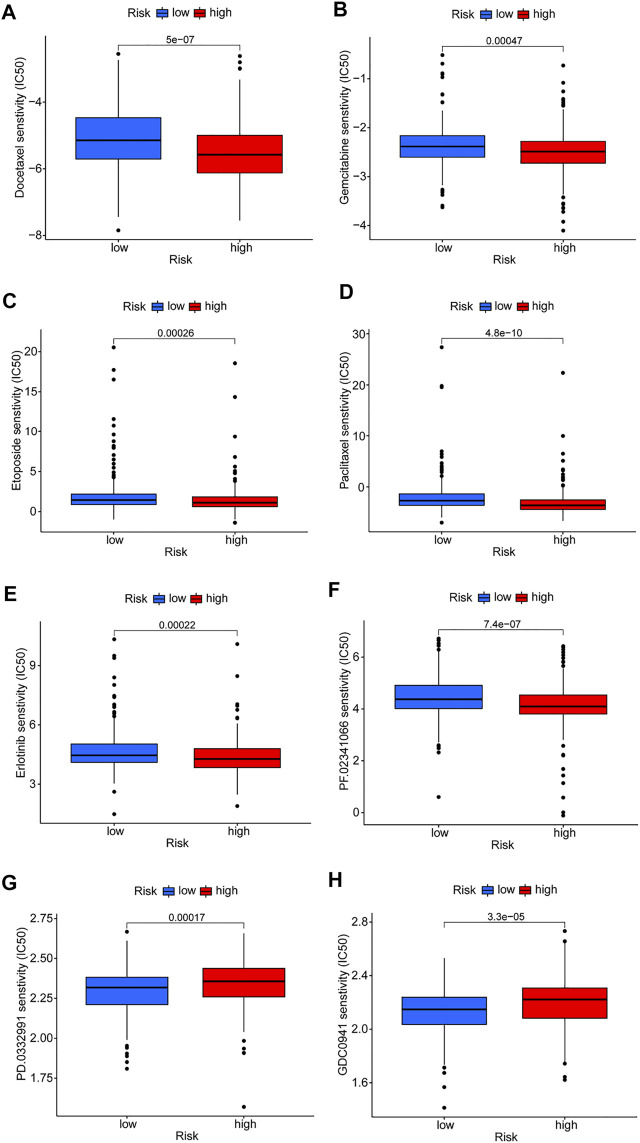
Prediction of drug susceptibility in different risk groups. **(A–F)** Sensitive drugs in high-risk groups. **(G,H)** Sensitive drugs in low-risk groups.

Differences in drug sensitivity of different risk subgroups were analyzed to investigate the clinical application value of the risk model. Results showed that docetaxel, paclitaxel, etoposide, gemcitabine, erlotinib, and crizotinib had good effects on patients in high-risk groups (Figures 8A–E). Patients in low-risk groups were more susceptible to drugs such as CDK4/6 inhibitors (PD.0332,991) and PI3K inhibitors (GDC0941); however, these drugs are currently used only for scientific research and may be promising in the future (Figures 8F,G). Reviewing the GSEA and DO enrichment analysis, it was found that the high-risk group in the TCGA-LUAD cohort had pathway enrichment such as cell cycle and DNA replication. The sensitive medications in the high-risk group are all first-line anti-tumor drugs for non-small cell lung cancer, among which chemotherapy drugs include docetaxel, paclitaxel, etoposide and gemcitabine, and their anti-tumor mechanisms are mainly directed against cell cycle and DNA replication. Erlotinib and crizotinib are two targeted medications, with erlotinib acting as an Epidermal Growth Factor Receptor (EGFR) inhibitor and crizotinib acting as an Anaplastic lymphoma kinase (ALK) inhibitor. Both EGFR and ALK targets are crucial for cancer proliferation. Presumably this is why the high-risk group is susceptible to the six antitumor drugs. The sensitive medications in the low-risk group have not been utilized in clinical practice. Fortunately, we discovered that the low-risk group had stronger immune infiltration and immune checkpoint expression, and it is expected that immunotherapy will be effective.

## Discussion

Numerous studies have recently revealed that m7G-related genes are closely linked to the development of cancer (Dai et al., 2021; Ma et al., 2021). A new class of lncRNAs has gradually become a research hotspot in various cancer fields in recent years. Some studies have found that abnormal expression of lncRNAs is associated with the occurrence and progression of LUAD and some lncRNAs may be highly correlated with prognosis (Spizzo et al., 2012; Cao et al., 2022; Xia et al., 2022). However, studies on m7G-related lncRNA predicting LUAD survival are scanty. The present study attempted to build a prognostic model of lncRNA in LUAD patients to test its clinical utility, and systematically explored the differences of risk models in immune cell infiltration, immune checkpoints, and drug sensitivity.

Forty publicly reported m7G-related genes were collected. First, lncRNAs that were differentially expressed in normal and LUAD tissues were explored. Univariate Cox regression was used to analyze the DELs, and 8 prognostic-related lncRNAs were screened out. Lasso-Cox regression analysis was then performed on these lncRNAs, and 6 prognosis-related lncRNAs (LINC01352, AP000695.1, AC018647.1, AL355472.3, AC026355.2, and SALRNA1) were finally identified and a risk prognosis model was constructed. The Sankey diagram showed that AC018647.1, AL355472.3, and SALRNA1 are related to DCP2. DCP2 is a decapping enzyme that plays a significant role in the regulation of the cell cycle and proliferation (Mugridge and Gross, 2018). DCP2 was found to promote lung cancer proliferation (Zhang et al., 2021). Our data also showed that AC026355.2 was highly correlated with four mRNAs (ACO2, DCP2, EIF4E, and NCBP1). Although AC026355.2 is rarely reported, we speculate that it plays a significant role in tumor development, but its precise role requires further investigation. EIF4E3 can promote translation, mRNA export, proliferation, and oncogenic transformation, and its related lncRNA LINC01352 was found to affect the growth and metastasis of hepatoma cells (Osborne et al., 2013). Bioinformatics analysis showed that AP000695.1 is closely related to immunity, and its related gene NUDT10, could be a potential immunotherapy target for LUAD in addition to promoting cell proliferation, inhibiting apoptosis, and causing tumor suppressor gene loss (Jin et al., 2020; Chen et al., 2021).

GSEA was performed on patients in both risk subgroups to reveal differences in biological function. Immune-related pathway enrichment was discovered in the low-risk group but not in the high-risk group. Immune cell bubble plots showed that low-risk groups had higher levels of immune infiltration. It has been reported that the high immune infiltration state tends to have a better immunotherapeutic effect (Guo et al., 2022; Luo et al., 2022). Interestingly, immune scores and immune checkpoint expression levels were also higher in the low-risk group, which is consistent with the results of Yu et al. (2021). Furthermore, susceptibility to antitumor drugs in different risk subgroups was assessed, and it found that the high-risk group showed high sensitivity to first-line treatment drugs for non-small cell lung cancer (including docetaxel, paclitaxel, etoposide, gemcitabine, erlotinib, and crizotinib) (Schiller et al., 2002; Zhou et al., 2011; Liang et al., 2017; Wu et al., 2018a; Wu et al., 2018b). Collectively, these results suggest that patients in the low-risk group will respond better to immunotherapy, while those in the high-risk group will respond better to chemotherapy and targeted drugs, which has important implications for individualized tumor therapy.

Although we verified the stability of the risk model from multiple aspects, there are still some limitations. First, the model was not externally validated because other databases lacked lncRNA information; thus, it was only be validated internally by TCGA. Further studies with a large sample size are required to draw definitive conclusions. Future studies will further explore the six lncRNAs.

## Conclusion

In summary, this study conducted a comprehensive bioinformatics analysis and developed a risk model for six m7G-related lncRNAs, which not only accurately predicts patient survival but also reflects the immune characteristics of LUAD patients. This may provide important clues for the development of clinical individualized treatments and promote the progress of immunotherapy.

## Data Availability

The original contributions presented in the study are included in the article, further inquiries can be directed to the corresponding authors.
